# Association between subclinical hypothyroidism and depression: an updated systematic review and meta-analysis

**DOI:** 10.1186/s12888-018-2006-2

**Published:** 2019-01-08

**Authors:** Huai Heng Loh, Lee Ling Lim, Anne Yee, Huai Seng Loh

**Affiliations:** 1 Faculty of Medicine and Health Sciences, University of Malaysia Sarawak(UNIMAS), Jalan Dato Muhammad Musa, 94300 Kota Samarahan, Sarawak Malaysia; 2 Division of Endocrinology, Department of Medicine, Faculty of Medicine, University of Malaya, Kuala Lumpur, Malaysia; 3 Department of Psychological Medicine, Faculty of Medicine, University of Malaya, Kuala Lumpur, Malaysia; 4 Clinical Academic Unit, Newcastle University Medicine Malaysia, Gelang Patah, Malaysia

**Keywords:** Subclinical hypothyroidism, Depression, Thyroid stimulating hormone, Levothyroxine

## Abstract

**Background:**

Although depression is associated with changes in the hypothalamic-pituitary-thyroid axis, its relationship with subclinical hypothyroidism (SCH) is controversial. To date, there is a lack of data on the improvement of depressive symptoms with levothyroxine therapy among individuals with coexistent SCH.

**Methods:**

We conducted a meta-analysis to evaluate the association between SCH and depression including 1) the prevalence of depression in SCH (with a sub-analysis of the geriatric cohort), 2) thyroid stimulating hormone (TSH) level among patients with depression and 3) the effect of levothyroxine therapy among patients with SCH and coexistent depression.

**Results:**

In a pooled analysis of 12,315 individuals, those with SCH had higher risk of depression than euthyroid controls (relative risk 2.35, 95% confidence intervals [CI], 1.84 to 3.02; *p* < 0.001). Geriatric cohort with SCH had a 1.7-fold higher risk of depression compared with healthy controls (odds ratio 1.72, CI, 1.10 to 2.70; *p* = 0.020). There was no difference in the mean TSH level between individuals with depression and healthy controls (2.30 ± 1.18 vs. 2.13 ± 0.72 mIU/L, *p* = 0.513). In individuals with SCH and coexistent depression, levothyroxine therapy was neither associated with improvement in the Beck Depression Inventory scoring (pooled d + = − 1.05, CI -2.72 to 0.61; *p* = 0.215) nor Hamilton Depression Rating Scale (pooled d + = − 2.38, CI -4.86 to 0.10; *p =* 0.060).

**Conclusion:**

SCH has a negative impact on depression. Early and routine screening of depression is essential to prevent morbidity and mortality. However, the use of levothyroxine among patients with SCH and coexistent depression needs to be individualized.

## Background

Neuropsychiatric disorders account for approximately 14% of the global burden of disease [1]. Depression, being one of the common chronically disabling disorders, can lead to poor quality of life [1, 2]. On the other hand, thyroid hormones (free triiodothyronine [fT3] and free thyroxine [fT4]) which are widely distributed in the central nervous system, regulate the neuronal growth and form synapses between neurons [3]. Given that depression is known to be associated with changes in the hypothalamic-pituitary-thyroid (HPT) axis [4], studies have reported its positive correlation with overt hypothyroidism [5]. However, its relationship with subclinical hypothyroidism (SCH) is not well established [6].

SCH is defined as an elevated thyroid stimulating hormone (TSH) with normal fT4 and fT3 levels. It affects 3 to 8.5% of the general population with a female preponderance, and a higher rate up to 20% among elderly people [7, 8]. This diagnosis is often overlooked especially when laboratory tests are not readily available, as these individuals with SCH tend to present with subtle and non-specific symptoms [9]. The debilitating effect of SCH on cardiovascular morbidity and mortality has gained increasing attention, suggesting that SCH is an independent risk factor for atherosclerotic cardiovascular disease [10, 11]. However, its association with depression remains controversial at large, with some studies indicated that SCH had the same propensity with overt hypothyroidism, while the others reported conflicting findings [7, 8, 12–24]. These inconsistent results could be attributed to the heterogeneous study populations, small sample size, lack of control arm for comparison and differences in study design.

To date, individuals with SCH are recommended to be initiated with levothyroxine replacement therapy only when their TSH level is above 10 mIU/L or if they are symptomatic, attempting pregnancy, have positive thyroid autoimmunity or cardiovascular risk factors, especially hypertension and hyperlipidemia [25, 26]. However, there is a lack of solid evidence in support of the use of levothyroxine therapy to improve mental health outcomes [27]. Hence, we performed an updated meta-analysis to evaluate the correlation between these two entities and the effect of levothyroxine therapy.

## Methods

We performed a systematic search of all English-language medical literature published from inception till June 2017 using PubMed, CINAHL and OVID electronic databases. We used MeSH headings of “subclinical hypothyroidism”, “thyroid”, “depression”, “thyroxine”, “geriatric” and “elderly”. We also reviewed references of the original article, reviews and clinical guidelines to identify additional eligible trials. Two independent reviewers (LHH and LLL) screened the titles and abstracts obtained through the electronic search and analysed the full-text articles. All duplicates were removed. Whenever needed, we contacted the authors to obtain either the full-text article or for clarification of the missing data. If the data were not provided numerically, it would be read off graphs. Two reviewers (LHH and LLL) extracted data from eligible studies independently using standard template, including authors, country of study conduct, study design, sample size, age, mean TSH levels, prevalence of major depression disorder (MDD) and the depression scores (based on the different depression scoring systems).

The analyses were divided into three parts. For part I, we examined the prevalence of depression in individuals with SCH compared with healthy controls. We also evaluated the mean depression scores among individuals with SCH, compared with their euthyroid counterparts based on the depression scales used. We performed a subgroup analysis on geriatric population, defined as those aged 60 years and above according to the United Nation’s classification [28]. For part II, we analysed the mean TSH level in individuals with depression compared with those without underlying psychiatric disorders. For part III, we evaluated the effect of levothyroxine replacement therapy on depressive symptoms among individuals with SCH.

## Study selection

For part I, we included studies which reported individuals who were diagnosed with SCH. Only case-control studies that either compared the prevalence of depression or the mean depression scores in SCH cohort with euthyroid/healthy controls were eligible. We excluded studies that included individuals who had been treated for thyroid disorders. Among the 15 studies included, there were nine questionnaires used to evaluate depression. Beck Depression Inventory (BDI) scoring and Hamilton Depression Rating Scale (HDRS) were the most commonly administered questionnaires, which were analysed in current meta-analysis.

For part II, we included only studies that assessed individuals with depression using standard depression assessment tools, such as the HDRS, Structural Clinical Interview for Diagnostic and Statistical Manual of Mental Disorders, Third Edition, Revised (DSM-III-R) and Fourth Edition (DSM-IV), and compared the mean TSH with those not known to have psychiatric disorders.

For part III, either randomized controlled trials or case-control studies that evaluated the effect of levothyroxine therapy in individuals with SCH and coexistent depression were included. Similar to part I, we analysed studies which used BDI and/or HDRS scoring only.

## Quality assessment

Two reviewers (LHH and LLL) independently appraised the quality of reporting of all included studies using the Newcastle-Ottawa Scoring (NOS) Scale for Case-Control Studies (Appendix). Any discrepancies were resolved by a third reviewer (LHS). The NOS scale was developed to assess the quality of non-randomized case-control studies for interpretation of meta-analysis results. It uses a “star system” which judges the studies in three broad categories, namely the selection of study group, group comparability and ascertainment of outcome of interest (“Exposure”). Each study can be awarded a maximum of one star for each numbered item (four in “Selection” category and three in “Exposure” category), and a maximum of two stars in the “Comparability” category. The maximum score is 10.

## Statistical analysis

All data analyses were performed using Stats Direct (version 2.7.9). Study characteristics were summarized. Descriptive statistics were shown as either mean ± standard deviation (SD), median (interquartile range, IQR) or number (percentages). We calculated the prevalence of depression in patients with SCH. The presence of heterogeneity between the trials was tested using the *I*^2^ statistic. An *I*^2^ value of more than 75% indicates significant heterogeneity. Due to the moderate and high heterogeneity (*I*^2^ ≥ 80% and ≥ 90%, respectively), data were pooled using the DerSimonian-Laird random-effects modelling. Pooled effect size (d+) was presented along with 95% confidence interval (CI) if the mean and SD of endpoint measures were reported in original articles. If the Cochrane Q test result was significant, we pooled the data using the DerSimonian–Laird random-effects modelling; otherwise, Hedges-Olkin fixed-effects modelling was used.

## Results

**Fig. 1:**
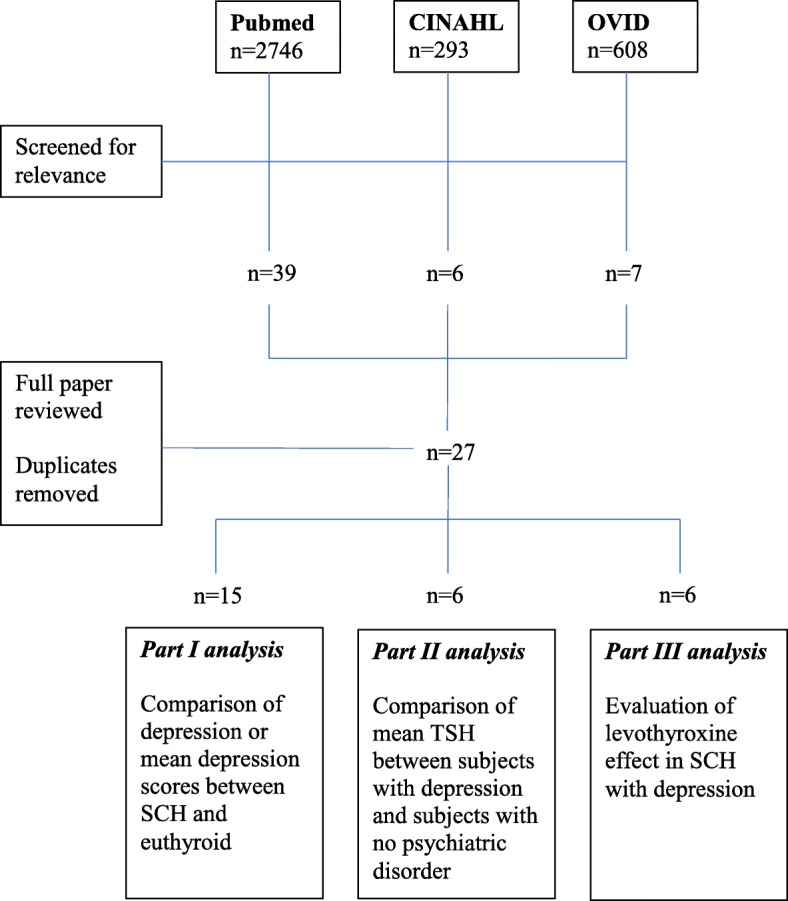
PRISMA search strategy

Figure 1 shows the flow of studies selection. Initial search identified 3647 articles and yielded 21 full-text articles after abstracts screening and duplicates removal. Out of these 21 articles, 15 were included in part I analysis, six into part II and III analyses, respectively.

### Part I - prevalence of depression in SCH

Table 1 summarizes the study characteristics. A total of 12,315 individuals (1700 [13.8%] with SCH) from 15 articles were included in this analysis. The sample sizes ranged from 22 to 8214. Six studies involved community-dwelling healthy individuals [12, 17, 20, 21, 23, 29] while the remaining nine single-centre studies recruited out-patients who were investigated for suspected SCH [7, 13–16, 19, 22, 24, 30]. Eight studies reported the mean depression scores between individuals with SCH and healthy controls, of which five used HDRS [12, 13, 15, 16, 22] and three used BDI [17, 23, 24]. All other studies used different but validated scales to diagnose depression.

**Table 1:** Characteristics of studies assessing prevalence of depression among patients with subclinical hypothyroidism

Author, year	Country	Setting	Study type	Depression Scale	SCH pts, n	M:F	Mean age (SD)	Mean TSH mIU/L (SD)	MDD, n	Non SCH Pts, n	M:F	Mean age (SD)	Mean TSH mIU/L (SD)	MDD, n	NOS scores
Haggerty 1993	USA	Community	Cross section	HDRS	16	0:16	36.3 (9.3)	4.6 (2.7)	9	15	0:15	34.2 (10.1)	1.7 (0.7)	3	8
Baldini 1997	Italy	Outpatient	Prospective	HDRS	19	0:19	55.2 (8.8)	12.0 (7.2)	NA	17	0:17	50.2 (9.2)	1.2 (1.2)	NA	9
Chueire 2002	Brazil	Outpatient	Cross section	SCI	112	NA	NA	NA	26	138	NA	NA	NA	16	7
Aslan 2005	Turkey	Outpatient	Cross section	HDRS	7	NA	39.2 (10.2)	93.8 (225)	1	15	NA	42.5 (11.0)	1.6 (1.1)	4	8
Gulseren 2006	Turkey	Outpatient	Prospective	HDRS	43	5:38	40.9 (14.2)	17.6 (2.16)	NA	20	NA	40.5 (7.6)	2.96 (0.96)	NA	9
Jorde 2006	Norway	Community	Prospective	BDI	89	45:44	62.4 (11.9)	5.72 (1.68)	NA	154	72:82	61.0 (12.5)	1.79 (0.69)	NA	9
Almeida 2006	Brazil	Outpatient	Cross section	HDRS	94	5:89	49.1 (10.3)	NA	24	43	3:40	44.8 (9.6)	NA	7	7
				BDI					43					9	
Park 2009	Korea	Community	Cross section	GDS-K	164	73:91	76.5 (9.0)	6.79 (3.77)	9	754	327:427	76.8 (9.0)	2.28 (0.91)	40	8
De Jongh 2011	Netherlands	Community	Cross section	CESD	64	20:44	74.9 (6.8)	6.89	11	1121	569:552	75.5 (6.5)	1.50	155	8
Demartini 2014	Italy	Outpatient	Cross section	HDRS	123	7:116	62.5 (13.4)	13.5 (14.9)	78	123	11:112	52.0 (14.7)	2.7 (1.0)	34	7
				MADRS					79					36	
Silva 2014	Brazil	Outpatient	Cross section	GDS-15	43	8:35	81.2 (6.7)	5.5 (1.2)	30	235	55:180	80.3 (6.8)	2.1 (0.9)	107	7
				CSDD											
Fjaellegaard 2014	Denmark	Community	Cross section	WHO-MDI	580	252:328	53	4.5	2	7634	3696:3938	53	1.7	3	8
Vishnoi 2014	India	Outpatient	Prospective	HDRS	300	92:208	52.5 (11.5)	7.8 (1.7)	185	300	87:213	52.4 (11.5)	2.8 (1.3)	NA	9
Krysiak 2016	Poland	Community	Cross section	BDI	17	0:17	31 (5)	7.5 (1.2)	10	16	0:16	30 (5)	2.6 (1.0)	6	9
Krysiak 2016	Poland	Community	Cross section	BDI	17	0:17	30 (4)	7.3 (1.3)	6	18	0:18	29 (4)	1.7 (0.6)	3	9
Krysiak 2017	Poland	Outpatient	Prospective	BDI	12	12:0	35 (6)	12.3 (4.0)	5	12	12:0	34 (7)	1.6 (0.8)	1	10

**Fig. 2:**
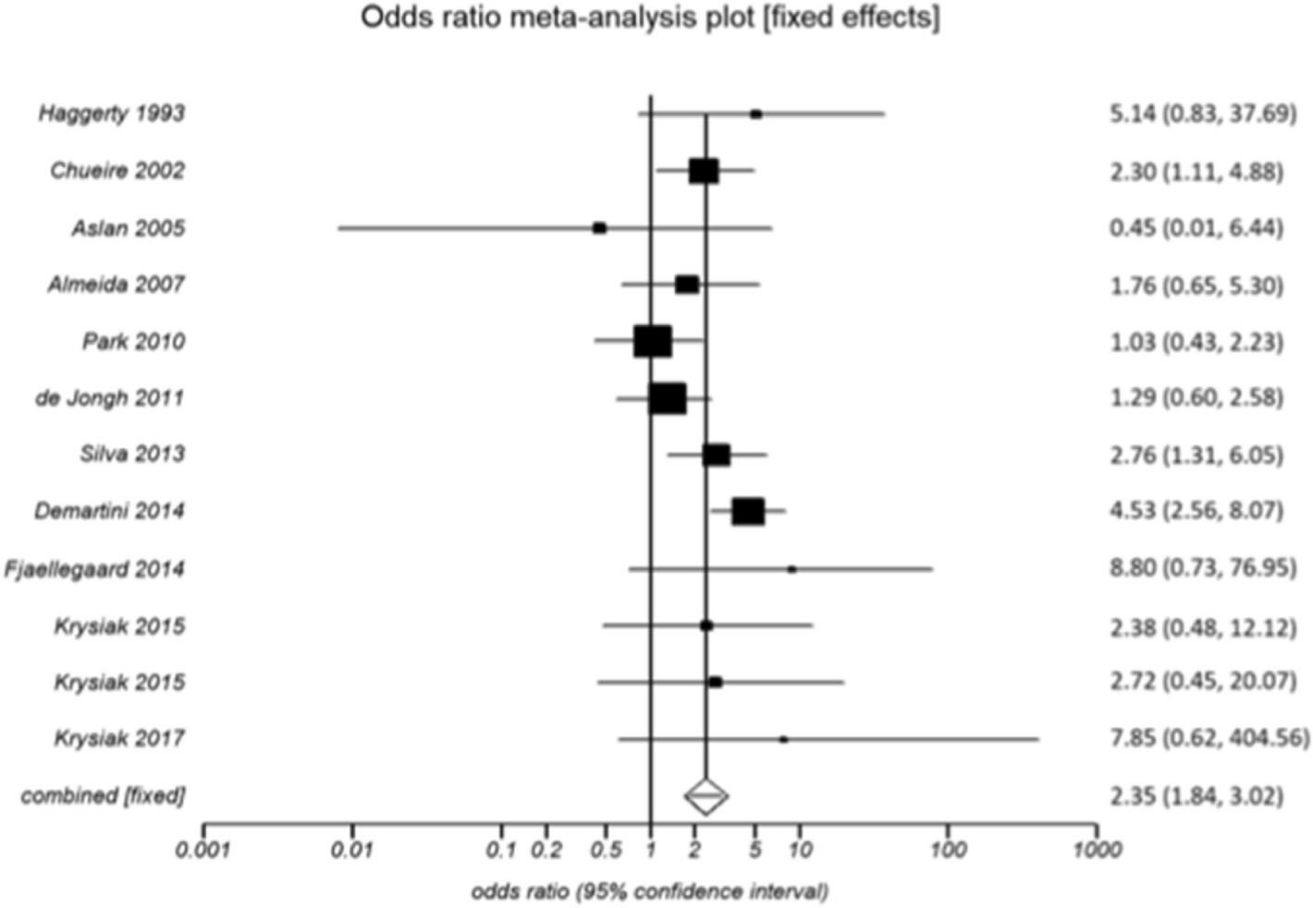
Risk of depression among patients with subclinical hypothyroidism

Overall, individuals with SCH were older compared with healthy controls (51.9 ± 17.5 vs. 50.2 ± 17.4 years, *p* = 0.020). The mean TSH in individuals with SCH was significantly higher than healthy controls (16.20 ± 24.75 vs. 2.09 ± 0.57 mIU/L; *p <* 0.001). Six studies found a higher prevalence of depression among individuals with SCH compared with controls [7, 12, 14, 19, 23, 24], although only three studies achieved statistically significant difference [7, 14, 30]. Five studies showed higher depression scores among patients with SCH than control group [12, 16, 22–24]. Five other studies showed no difference in the number of individuals with depression between SCH and control group [8, 15, 20, 21, 30], whereas three found no difference in the depression scores [13, 15, 17]. However, pooled analysis showed that individuals with SCH had higher risk of depression compared with euthyroid controls (relative risk 2.36, 95% CI 1.84 to 3.02; *p* < 0.001) (Fig. 2). Compared with healthy controls, there was no significant difference in the depression scores among individuals with SCH, using either BDI scoring (10.85 ± 4.97 vs. 8.1 ± 2.82; *p =* 0.126) or HDRS scale (8.02 ± 2.44 vs. 5.66 ± 2.59; *p* = 0.312, 95% CI -2.22 to 6.70).

**Fig. 3:**
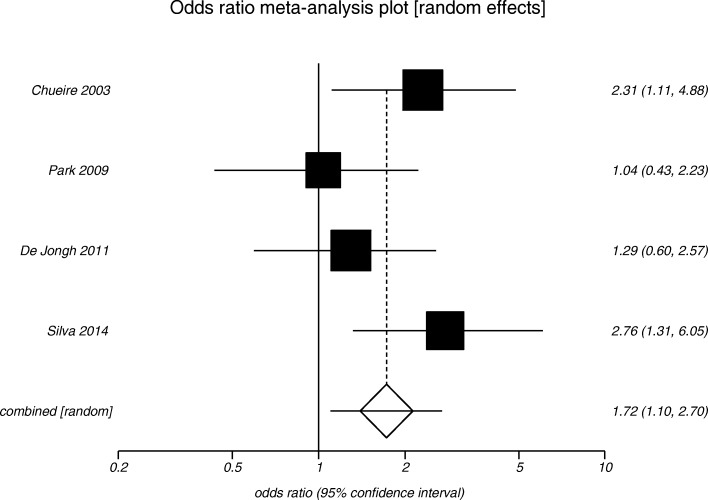
Risk of depression among geriatric patients with subclinical hypothyroidism

In geriatric cohort with SCH, four studies evaluated the prevalence of depression [8, 14, 20, 30], of which two recruited eligible individuals from single centre out-patient clinics [14, 30] and another two involved community-dwelling healthy controls [8, 20]. Their mean age did not differ from the euthyroid controls (77.5 ± 3.3 vs. 77.5 ± 2.5 years; *p* = 0.770). The mean TSH was higher among SCH individuals than euthyroid controls (6.15 ± 0.91 vs. 2.19 ± 0.13 mIU/L; *p* < 0.001). Two studies found a higher prevalence of depression between elderly with SCH compared to controls [14, 30]. When we pooled the data from these four studies, elderly individuals with SCH had a 1.7-fold higher risk of depression than healthy controls (odds ratio 1.72, 95% CI 1.10 to 2.70; *p* = 0.020) (Fig. 3).

### Part II - TSH level in depression

Table 2 shows the characteristics of seven cross-sectional articles involving 7135 individuals, of whom 4942 (80.3%) were men. Three studies recruited individuals who were hospitalized for psychiatric disorder without prevalent diseases such as metabolic, autoimmune and other endocrine diseases, infections and inflammatory disorders [31–33]. Two studies involved individuals seen in the psychiatric out-patient clinics [34, 35], three from psychiatric wards [31–33] whereas the other two studies selected individuals from the elderly community [36, 37]. All studies reported mean TSH of the study cohort. Five studies reported the prevalence of SCH among those diagnosed with depression [31–33, 35, 36].

**Table 2:** Characteristics of studies assessing TSH level in depression

Author, year	Country	Setting	Depression Scale	MDD pts, n	M:F	Mean age (SD) or age range	SCH pts, n	Mean TSH mIU/L (SD)	Non MDD pts, n	M:F	Mean age (SD) or age range	Mean TSH mIU/L (SD)	NOS score
Boral 1980	India	Out-patient	NA	31	16:15	22–52	NA	4.58 (1.17)	31	16:15	22–52	3.07 (0.334)	8
Maes 1993	Belgium	In-patient	HDRS	57	20:37	54.2 (1.7)	0	0.99 (0.1)	69	33:36	40.8 (1.4)	1.67 (0.11)	8
Custro 1994	Italy	In-patient	BDI	9	0:9	30–69	5	1.73 (0.3)	38	0:38	49.9	1.17 (0.05)	7
Vandoolaeghe 1997	Belgium	In-patient	HDRS	36	20:16	50.3 (14.0)	1	1.44 (1.02)	15	10:5	47.5 (15.0)	1.73 (0.99)	8
Saxena 2000	India	Out-patient	NA	32	21:11	36.2 (11.8)	6	3.51 (1.78)	11	5:6	30.54 (12.6)	3.27 (0.97)	8
Almeida 2011	Australia	Community	GDS	189	189:0	75.9 (4.3)	17	2.2 (1.3)	3742	3742:0	75.2 (4.1)	2.3 (2.0)	8
Saltevo 2015	Finland	Community	BDI	236	0:236	61 (9)	NA	1.97 (1.38)	1114	0:1114	59 (8)	1.92 (1.18)	8
Saltevo 2015	Finland	Community	BDI	163	163:0	62 (9)	NA	1.94 (1.25)	1136	1136:0	60 (8)	1.87 (1.24)	8

Among 753 (10.6%) individuals with depression, 429 (57.0%) were men. The mean age was higher among individuals with depression than healthy controls (56.6 ± 13.3 vs. 52.2 ± 15.9 years; *p* = 0.180). There was no difference in mean TSH between individuals with depression and healthy controls (2.30 ± 1.18 vs. 2.13 ± 0.72 mIU/L; *p* = 0.513).

### Part III - effect of levothyroxine on depressive symptoms

Six papers which involved a total of 266 individuals assessed the improvement of depression scores with levothyroxine therapy in the SCH cohort. Three papers utilized BDI scoring [17, 24, 38], whereas the remaining three used HDRS [13, 16, 22].

**Fig. 4:**
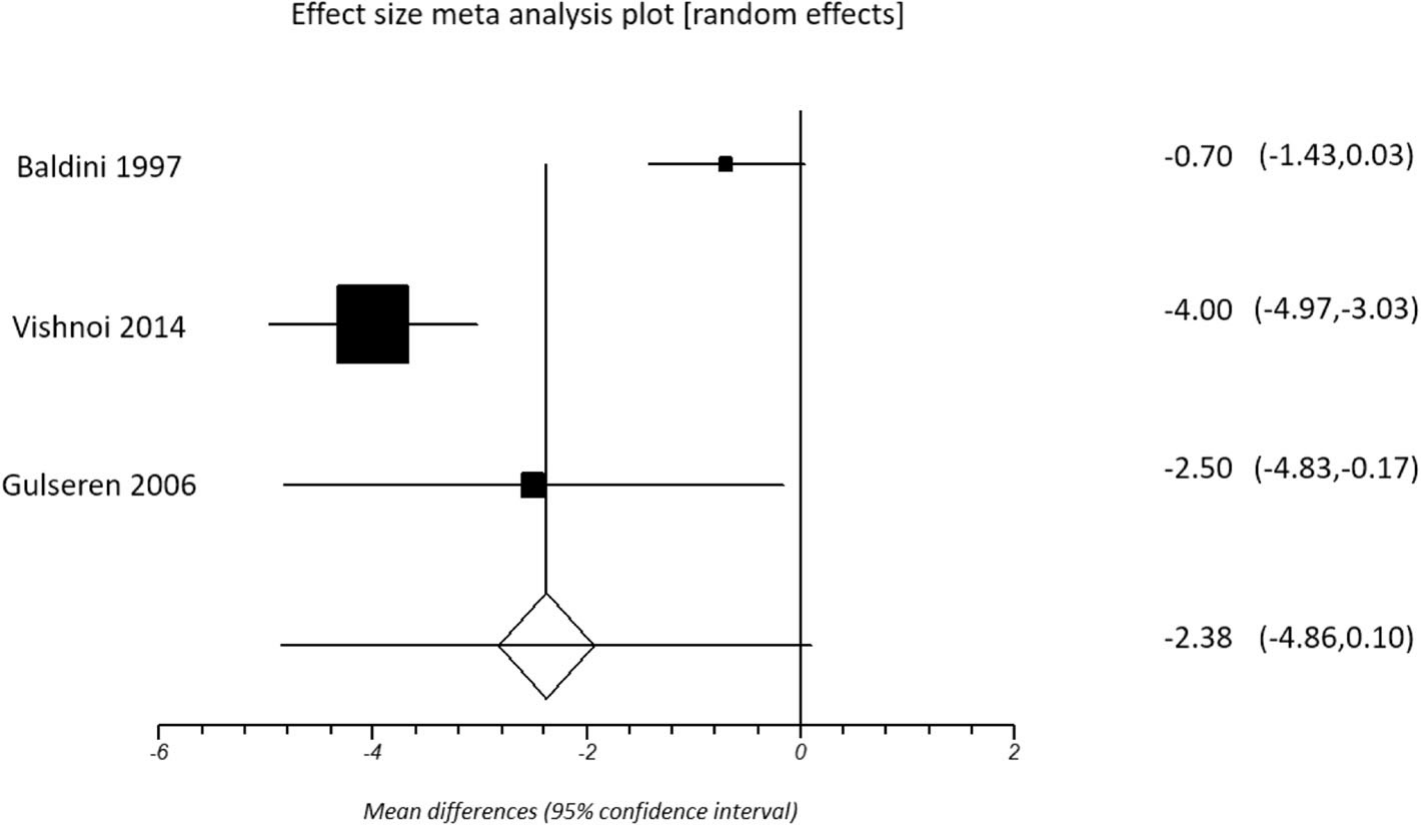
Changes in BDI scores with levothyroxine treatment

**Fig. 5:**
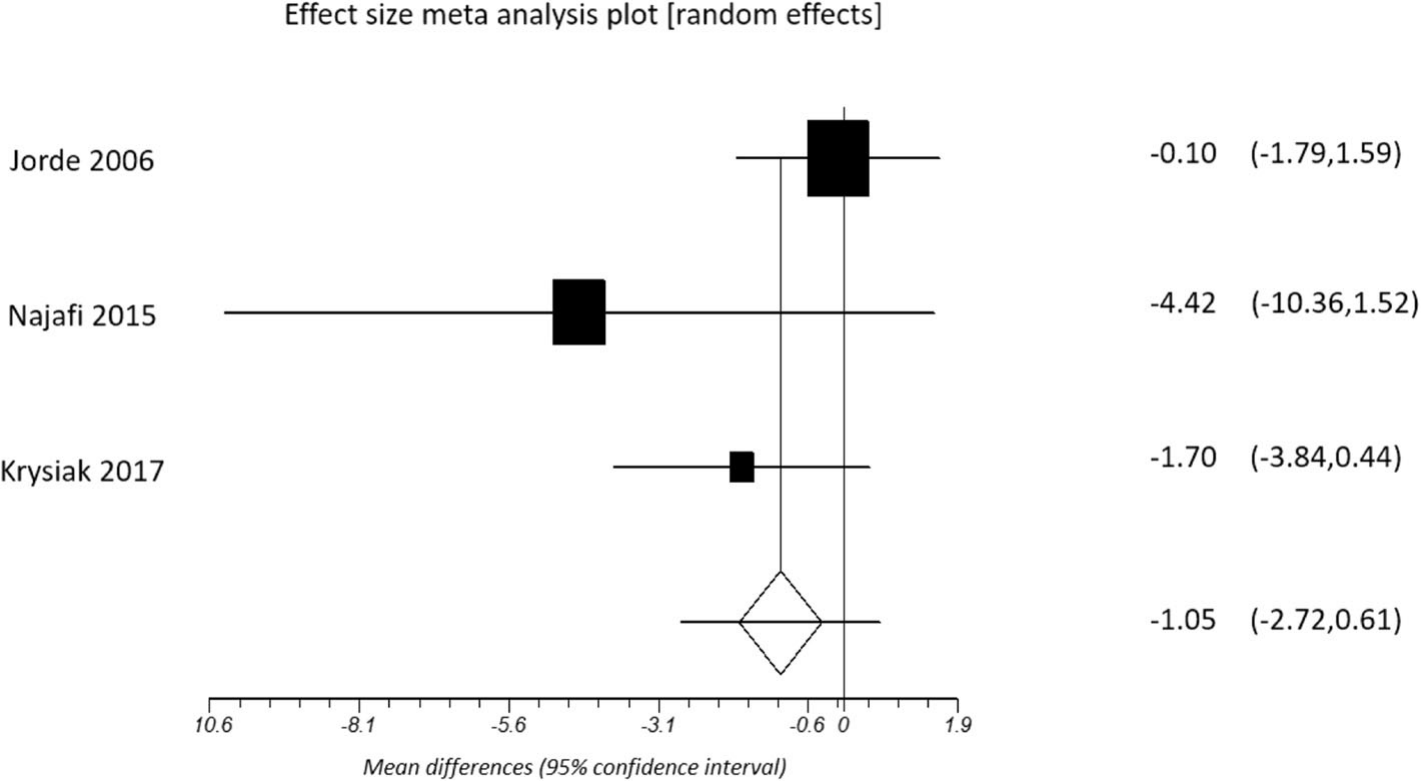
Changes in HDRS scores with levothyroxine treatment

The duration of intervention with levothyroxine therapy in these studies ranged from 2 to 12 months, with a mean duration of 5.95 ± 4.09 months. All but one study [17] reported a significant improvement in TSH with levothyroxine therapy (pooled d + = − 8.09, 95% CI -12.56 to − 3.63; *p* < 0.001). Three studies showed significant reduction in depression scores, from 10.3 ± 4.6 pre-treatment to 6.3 ± 3.4 post treatment, *p* = 0.00 (HDRS) [22], from 8.3 ± 5.2 pre-treatment to 5.8 ± 4.9 post-treatment *p* < 0.05 (HDRS) [16], and from 16.79 ± 13.25 pre-treatment to 12.37 ± 10.01 post-treatment, *p* = 0.04 (BDI) [38]. However, pooled analysis using random-effects modelling revealed no significant improvement in either BDI scores (pooled d + = − 1.05, 95% CI -2.72 to 0.61; *p* = 0.215) (Fig. 4) or HDRS (pooled d + = − 2.38, 95% CI -4.86 to 0.10; *p =* 0.060) (Fig. 5).

## Discussion

Present updated meta-analysis of 21 studies reported nearly 2.5-fold excess risk of depression in adults with SCH, particularly in elderly population, compared with their euthyroid counterparts. Our findings can potentially influence the clinical practice and improve the quality of life of patients.

The association between mood disorders and changes in the HPT axis has been recognized, although this is frequently reported in individuals with overt hyper−/hypothyroidism [5, 39–41]. In the central nervous system, alterations in hormones levels such as somatostatin and serotonin, can result in neuropsychiatric disturbances [42]. Existing evidence also suggests that these mechanisms can potentially affect the HPT axis and thus, explain the association between SCH and depression. Several studies reported a reduction in the somatostatin level in cerebrospinal fluid, leading to increased TSH level among individuals with depression [43, 44]. On the other hand, serotonin deficiency, which is also commonly seen in those with depression, has been postulated to cause alterations in the HPT axis [45]. Taken together, SCH and depression may share common biological mechanisms, which is in support of our findings.

Traditionally, depression is reported among people aged 35–45 years [46]. Of note, it has become increasingly common in the elderly as normal aging itself is associated with biochemical changes in the HPT axis. The secretion of thyroid hormones is reduced with increasing age, with a lower fT3 level but a relatively unchanged fT4 concentration. Compared with the younger population, higher TSH level is seen in the elderly due to reduced fT4 degradation and its peripheral conversion to fT3, with subsequent positive feedback to the HPT axis [47]. Individuals with overt hypothyroidism experience a wide variety of clinical signs and symptoms including cold intolerance, weight gain, cognitive dysfunction and mood disturbances [48, 49]. It is important to note that only up to 30% of individuals with SCH share similar clinical features [50], with the elderly group experience even fewer and more subtle complaints, which results in a delayed diagnosis of SCH [51]. Given the world population ageing and an increased risk of depression in the elderly with SCH as shown in our analysis [52], there is an urgent need for TSH and depression screening in this vulnerable population, in order to improve health and well-being for all.

Although our findings showed no significant difference in the serum TSH level between individuals with depression and healthy controls, this could possibly be a phenomenon known as the “brain hypothyroidism” [6], which represented a low intracerebral fT3 concentration with normal peripheral thyroid hormones and TSH levels [53]. Physiologically, type II deiodinase converts fT4 to fT3 in the brain glial cells [54]. However, it has been postulated that depression can cause inhibitory effect on type II deiodinase, which leads to the conversion of fT4 to rT3 via type III deiodinase [55]. Furthermore, transthyretin, a serum transport protein for fT4 in cerebrospinal fluid, is reduced in individuals with refractory depression [53]. Ultimately, these result in decreased intracerebral fT3 and fT4 levels, along with a high rT3 concentration in cerebrospinal fluid that can also inactivate fT3 activity [55, 56]. Nevertheless, further studies which examine the variations between intracerebral and peripheral thyroid hormones and TSH levels in different populations are required to allow a better understanding of this complex relationship.

Our meta-analysis did not show improvement in the symptoms of depression with the use of levothyroxine therapy among individuals with coexistent SCH. There are several possible explanations. Of the six studies included, the duration of intervention was relatively short and heterogenous (mean: 6 months, range: 2–12 months), which might contribute to the differential effects seen with levothyroxine therapy. On the other hand, given a reduced activity of intracerebral type II deiodinase in depression, levothyroxine therapy might be converted to rT3 that could exacerbate the existing intracerebral fT3 deficiency. In a seminal series of nine individuals with refractory depression, on top of antidepressant and levothyroxine therapy, additional administration of liothyronine was associated with marked improvement in the symptoms of depression in seven of them [57]. To date, the evidence related to the effect of normalization in thyroid hormones and TSH level in depression is inconclusive, perhaps due to the type of thyroid hormone replacement therapy used (single or combination), severity of SCH and depression, timing and duration of intervention.

To the best of our knowledge, our report has explored further in details the relationship between SCH and depression, by including the geriatric cohort and examining the effect of levothyroxine therapy in individuals with these co-existing illnesses. Importantly, our analysis indicated a clear negative impact of SCH in depression. However, our study has a few limitations. Meta-analyses are known to be confounded by the comprehensiveness of search strategy, reporting quality of included studies, publication bias and exclusion of non-English articles. In this analysis, explicit criteria were applied in our extensive literature search and investigators were contacted for clarification whenever necessary. Besides, the study populations were considerably older with a mean age of 52 years, which could limit the generalizability of our results.

## Conclusions

Our findings favor early and routine screening for depression among individuals with SCH especially the elderly, to prevent morbidity and mortality. However, the use of levothyroxine therapy among people with depression need to be considered carefully on an individual basis, weighing the risks and benefits of the treatment. More well-designed population-based prospective studies or randomized controlled trials are needed to gain more insights of the pathogenesis and natural history of depression in SCH, as well as the efficacy and safety of levothyroxine therapy to improve mood disorders among these high-risk individuals of varying age groups.

## Abbreviations

BDI: Beck depression inventory
DSM-III-R: Structural clinical interview for diagnostic and statistical manual of mental disorders, third edition, revised
DSM-IV: Structural clinical interview for diagnostic and statistical manual of mental disorders fourth edition
fT3: Free triiodothyronine
fT4: Free thyroxine
GDS: Geriatric depression scale
HDRS: Hamilton depression rating scale
HPT: Hypothalamic-pituitary-thyroid
MDD: Major depression disorder
NA: Not available
NOS: Newcastle-ottawa scoring
SCH: Subclinical hypothyroidism
TSH: Thyroid stimulating hormone
